# The Role of HK2 in Tumorigenesis and Development: Potential for Targeted Therapy with Natural Products

**DOI:** 10.7150/ijms.105553

**Published:** 2025-01-21

**Authors:** Keren He, Fangfang Tao, Yangyuxiao Lu, Mengqi Fang, Hong Huang, Yuan Zhou

**Affiliations:** 1 The First Affiliated Hospital of Zhejiang Chinese Medical University, Hangzhou, China.; 2 School of Basic Medical Sciences, Zhejiang Chinese Medical University, Hangzhou, China.; 3 Zhejiang Key Laboratory of Blood-Stasis-Toxin Syndrome, Zhejiang Chinese Medical University, Hangzhou, China.

**Keywords:** Hexokinase 2, Glycolysis, Anticancer, Natural products, Therapeutic target

## Abstract

Hexokinase 2 (HK2) is widely distributed in various tissues, particularly showing significantly elevated expression levels in tumor tissues. As the initial rate-limiting enzyme in the glycolysis process, HK2 is believed to directly participate in the metabolic reprogramming of tumor cells. This phenomenon, known as the “Warburg effect,” provides the energy and substances necessary for the rapid proliferation, growth, and division of tumor cells. Furthermore, by enhancing glycolysis, HK2 exerts its influence on various metabolic pathways in tumor cells, such as pentose phosphate metabolism, glutamine metabolism, serine metabolism, and glycine metabolism, thereby playing a role in the occurrence and development of cancer. Therefore, HK2 represents a promising target for cancer therapy. Simultaneously, natural products with effects on inhibiting the expression or activity of HK2, have already been discovered to exhibit significant anticancer potential. Flavonoids, pentacyclic triterpenoids, phenolic compounds, and lignans constitute the majority of these natural products, directly inhibiting HK2 or indirectly downregulating it through protein kinase B (AKT), hypoxia-inducible factor 1 alpha (HIF-1α), and c-Myc signaling pathways. However, several challenges remain, such as further screening for natural products that directly target and inhibit HK2, optimizing the selection of natural product inhibitors for HK2, and elucidating the molecular mechanisms by which natural products indirectly inhibit HK2. In conclusion, the potential of targeting HK2 for cancer therapy is promising, and with these challenges addressed, natural products inhibiting HK2 will play an even greater role in the fight against cancer.

## 1. Introduction

The Hexokinase (HK) protein family is one of the central regulators of the glycolysis pathway, which converts D-hexose to D-hexose 6-phosphate and directly affects cellular glycolysis and glucose utilization. In mammals, the HK family can be divided into four isoenzymes encoded by different genes: HK1, HK2, HK3, and HK4 1. The molecular weights of HK1, HK2, and HK3 are all 100 kDa. HK1 is highly expressed in the brain and thus known as “cerebral hexokinase” 2. HK2 is highly expressed in tissues with high energy requirements such as liver, skeletal muscle and brain tissue 3. HK3 is predominantly expressed in spleen and lymph cells 4. HK4, also known as glucokinase, has a molecular weight of 50 kDa and regulates the generation of sugar and insulin release in the liver and pancreatic beta cells, which is crucial for controlling blood sugar levels throughout the body 5.

The total length of HK2 gene is about 50 kb, composed of 18 exons, encoding a protein with a molecular weight of 100 kDa and 917 residues, whose N-terminal and C-terminal are highly similar, and has certain catalytic activity 6. As a member of the HK family, HK2 can catalyze the conversion of glucose into glucose 6-phosphate (G6P), effectively preventing glucose from leaving the cell. It was discovered that HK2 promoted glycolytic pathways more quickly than other subtypes 7. Typically, HK2 binds to the voltage dependent anion channel 1 (VDAC1) protein on the mitochondrial outer membrane, thereby utilizing mitochondrial ATP for G6P production 8. As a pivotal member, VDAC1 primarily regulates substance exchange, cell death, and mitochondrial permeability 9.

At the same time, it was found that HK2 was highly expressed in cancer cells and played an important role in the “Warburg effect” (metabolic reprogramming from oxidative phosphorylation to glycolysis) 10. This phenomenon was first observed by Otto Warburg about 100 years ago 11. Even when cells have sufficient oxygen, they still adopt the glycolytic metabolic pathway, introducing glucose into the cytoplasm and, with the aid of pyruvate kinase and lactate dehydrogenase, converting it into lactic acid and nicotinamide adenine dinucleotide (NAD+) instead of further oxidizing it to carbon dioxide and water 12, 13. Therefore, HK2 is believed to be a key factor in promoting tumor growth and therefore stands out among the HK family and is currently the subject of extensive research. Natural products have gained widespread attention in cancer treatment due to their broad availability and high safety profile. Some compounds have also shown potential in inhibiting HK2-mediated enhancement of glycolysis in tumor cells. This article summarizes the role of HK2 in regulating tumor metabolic reprogramming and presents advancements in anticancer research of certain natural HK2 inhibitors. In conclusion, HK2 represents a promising therapeutic target for cancer treatment.

## 2. Expression of HK2 in normal and tumor cells

The expression of HK2 shows some differences in primary cells lines. HK2 is widely distributed in many primary cells, but usually exhibits low basal level expression. In the metabolism of active groups such as insulin sensitive tissues or specific physiological conditions, HK2 may show high level 14. Taking liver and brain as representatives, HK2 exhibits high expression to meet the energy demands of these tissues 3, 15. In addition, the expression level of HK2 in muscle tissue (another high energy demand tissue) is also relatively high, and HK2 can assist in the phosphorylation of intracellular glucose to provide energy for muscle movement. In skeletal muscle cells, HK2 is involved in the process of skeletal muscle cell contraction recovery, which syntheses glucose into glycogen to restore energy reserves 1. In adult cardiomyocytes, HK2 dynamically shuttles between mitochondria and cytoplasm in response to changes in intracellular G6P content, pH, and the cardioprotective signaling pathway AKT (protein kinase B) 16. At the same time through the combined with mitochondria, HK2 can balance the energy metabolism of cells, prevent myocardial cell apoptosis phenomenon, so as to protect the heart health 17. In general, the expression of HK2 in different cell types showed some differences and was usually closely related to the energy requirements and glucose metabolic status of the cells. This variation reflects the functional diversity of HK2 in different cell types and its importance in maintaining normal cellular metabolic balance.

It is noteworthy that research has revealed the phenomenon of high expression of HK2 in many cancer tissues or cells, such as colorectal cancer 18, brain metastases tumor 19, and pancreatic cancer 20. Hepatocellular carcinoma (HCC) cells exhibit high affinity for HK2 in metabolism and inhibit the expression of glucokinase. After silencing HK2 in human HCC cells, the occurrence of tumor was inhibited and the apoptosis of tumor cells was promoted 21. Additionally, compared with normal tissues, breast cancer samples exhibited significantly increased expression of HK2. And the absence of HK2 prevented breast cancer from metastasizing 22. As a glucose sensor and protein kinase, HK2 plays an important role in the regulation of tumor immune escape. In human glioblastoma cells, high glucose stimulates the separation of HK2 from mitochondria and binds to and phosphorylates Inhibitor of nuclear factor kappa-B alpha (IκBα) at T291A. This promotes the degradation of IκBα by the μ-calpain protease, thereby allowing nuclear factor kappa B (NF-κB) to be released from IκBα binding and translocated into the nucleus, enhancing Programmed death-ligand 1(PD-L1) transcription and promoting tumor immune escape 23. Due to the heightened dependency of cancer cells on glucose, the overexpression of HK2 contributes to meeting the high-energy state required for the rapid proliferation of cancer cells. Therefore, HK2 not only plays a role in maintaining normal cell metabolism but also serves as a crucial factor in promoting tumor growth. HK2 has also emerged as a potential target for anticancer drugs, garnering increasing attention.

## 3. HK2-mediated glycolysis enhancement in tumor cells

In order to meet the energy and biosynthesis requirements of their rapid growth, rapid proliferation, and other biological activities, tumor cells undergo glucose metabolic reprogramming. Normally, the vast majority of cells use the oxidative phosphorylation pathway to convert glucose into energy. However, through the reprogramming of glucose metabolism, cancer cells are more inclined to choose the aerobic glycolysis pathway. Even in an oxygen-rich environment, tumor cells metabolize glucose into lactic acid, which is also known as the Warburg effect.

Glucose metabolic reprogramming provides the following advantages to cancer cells. Firstly, by metabolic reprogramming, tumor cells can obtain the intermediates in the biosynthesis pathway, such as RNA, glycerin, and non-essential amino acids 24. At the same time, a large amount of lactic acid can be transported to the cell through the monocarboxylate transporter, resulting in acidity of the surrounding environment, contributing to the immune escape of tumor cells, and promoting their growth, spread and metastasis 25. HK2, as the foremost rate-limiting enzyme in glycolysis, exhibits high expression in numerous tumor cells and plays a pivotal role in the enhanced glycolysis of tumors.

The reprogramming of glucose metabolism in tumor cells reflects changes on multiple levels, first, increased glucose uptake and utilization capacity. The surface of tumor cells typically exhibits an overabundance of glucose transporters, such as Glucose transporter 1 (GLUT1), facilitating increased glucose uptake by the cells 26. Furthermore, tumor cells express the glycolytic enzyme pyruvate kinase (PK-M2) in a selective manner. This limits the conversion of phosphoenolpyruvate to pyruvate, increases the production of lactic acid, and decreases the amount of oxygen consumed. This better supports the metabolic activities necessary for cell proliferation, allowing for unrestricted growth and continuous proliferation 27.

The reverse Warburg effect is another unique metabolic process that occurs in the tumor microenvironment (TME) 28, 29. During this process, stromal cells in the tumor microenvironment, such as tumor-associated fibroblasts (CAFs), undergo metabolic reprogramming, shifting from an oxidative phosphorylation metabolic state to a glycolytic metabolic state 30, 31. In the "two-compartment" model of the reverse Warburg effect, cancer cells secrete hydrogen peroxide to induce oxidative stress of CAFs, prompting CAFs to produce a large number of metabolites such as lactic acid and pyruvate through aerobic glycolysis. Surrounding tumor cells can take up these metabolites and use them as energy substances or for biosynthesis, thereby promoting their own growth and proliferation 30, 32. HK2 can affect the cell viability, proliferation, invasion and migration of CAFs, as well as the glycolysis function of CAFs, thus affecting the production of lactic acid and the proliferation of surrounding cancer cells 28, 33.

### 3.1. The regulation of HK2 by oncogenes and tumor suppressor factors

**Figure 1:**
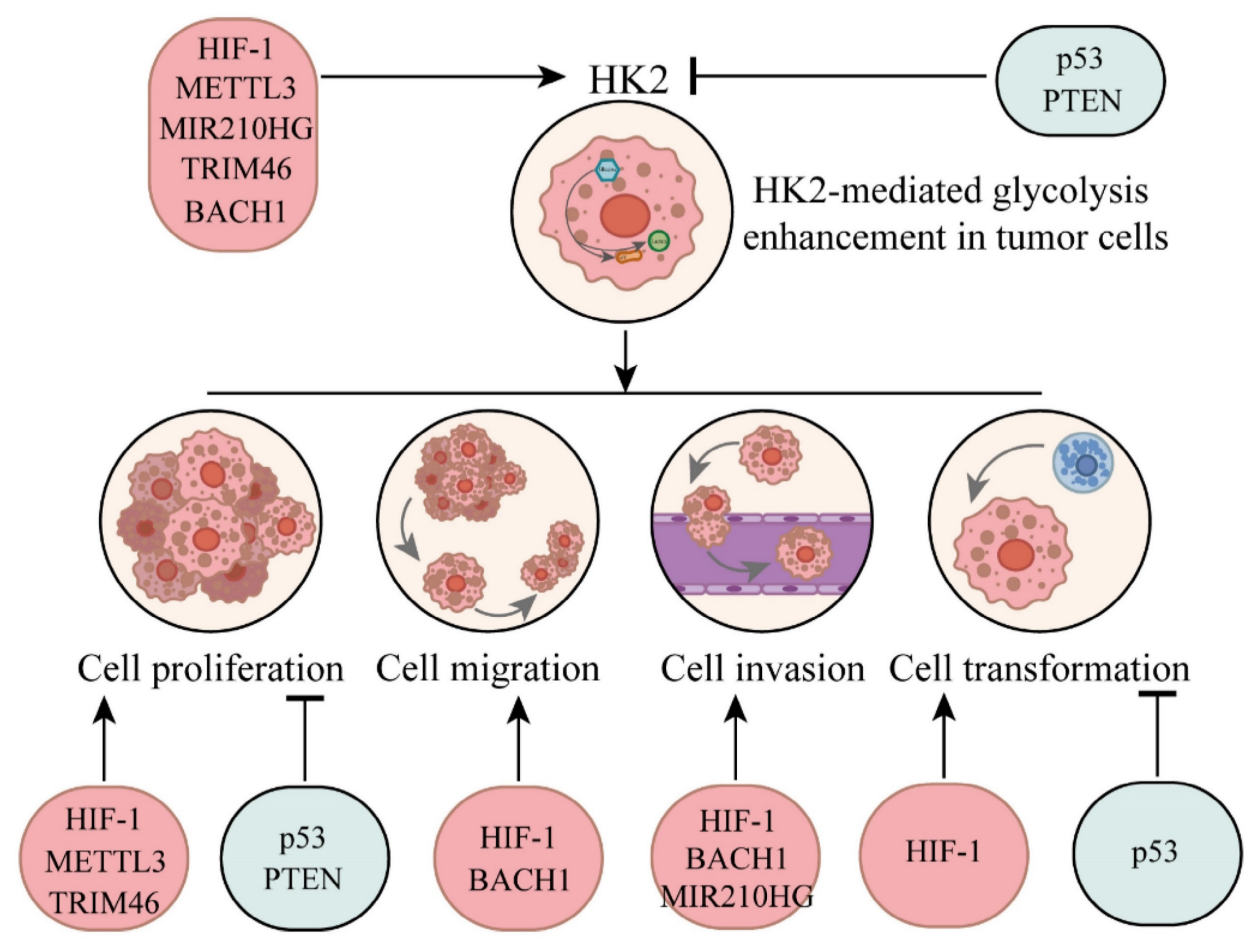
Upstream regulatory factors influence HK2 and its impact on tumor progression through regulating HK2.

Dysregulation of oncogenes and tumor suppressors usually leads to changes in the expression level of HK2, which in turn affects the reprogramming of glucose metabolism and has an important impact on the survival and proliferation of cancer cells **(Fig. 1)**. As an N^6^-Methyladenosine (m^6^A) methyltransferase, methyltransferase-like 3 (METTL3) is mostly reported as an oncogene, which can promote the occurrence and development of hematopoietic malignancies and solid tumors 34. METTL3 has been shown to stabilize HK2 expression in colorectal cancer through an m^6^A-IGF2BP2/3 dependent mechanism, thereby further regulating glycolytic metabolism and cell proliferation 35. Moreover, m^6^A modification mediated by YTH domain-containing family protein 1 (YTHDF1) increased the stability of HK2 and promoted the Warburg effect of cervical cancer cells 36. Hypoxia-inducible factor 1 (HIF-1) is a key regulator of metabolic reprogramming that occurs in hypoxic cancer cells 37. Studies have found that HIF-1α could combine with HK2 promoter sites, raise the expression of HK2 to promote cancer cell to glucose metabolic reprogramming 38.

MIR210 host gene (MIR210HG), as an important oncogene, plays a role in promoting the occurrence, development and metastasis of cancer. It was found that MIR210HG was positively correlated with HK2, and mechanism study revealed that MIR210HG promoted the expression of HK2 39. Tripartite motif containing 46 (TRIM46), is an oncogene that promotes proliferation of breast cancer cells *in vitro* and enhances tumor growth *in vivo* 40. Studies have found that overexpression of TRIM46 increased HK2 level, while knockdown decreased expression of HK2 41.

BTB domain and CNC homolog 1 (BACH1) is a transcriptional regulatory factor, which is closely related to tumorigenesis and progression. It is involved in regulating gene expression in tumor cells, affecting cell proliferation, metastasis, apoptosis and other important processes. BACH1 is also involved in cellular response to oxidative stress and is associated with iron metabolism and heme synthesis 42. Studies have shown that antioxidants reduced the level of free haem in cancer cells, leading to the stabilisation of BACH1. BACH1 binds to the HK2 and Glyceraldehyde 3-phosphate dehydrogenase (GAPDH) promoters, stimulating the expression of both and several other glycolytic genes, thus increasing glucose uptake, glycolysis rate and lactic acid secretion, thereby stimulating lung cancer cell metastasis 43. Targeting BACH1 could normalise glycolysis and prevent breast cancer cell metastasis, while overexpression of BACH1 could enhance glycolysis and promote breast cancer cell metastasis 43.

In contrast, the regulation of HK2 by tumor suppressors is usually negative. Phosphatase and tensin homolog (PTEN) and p53 are important tumour suppressor genes, and when they are both missing in prostate cancer, the expression of the HK2 gene is selectively increased 44, 45. In Oral squamous cell carcinoma (OSCC) cells, overexpression of PTEN can inhibit the activation of AKT, reduce the expression of GLUT1 and HK2, and thereby inhibit glycolysis and cell proliferation 46. Some studies have also found that in the absence of PTEN, Phosphoinositide 3-kinases (PI3K) catalysed the production of Phosphatidylinositol-3,4,5-trisphosphate (PIP3), and PIP3 bound to the pleckstrin homology domain of AKT, thereby activating AKT 47. Downstream of the PI3K-AKT kinase pathway, mechanistic target of rapamycin (mTOR) assembers with regulatory proteins to form mechanistic target of rapamycin complex 1 (mTORC1), phosphorylates S6 kinase 1(S6K1) and eukaryotic translation initiation factor 4E-binding protein 1 (4EBP1), and increases the synthesis of various proteins including HK2 protein, thereby promoting cell survival, proliferation and growth 47. AKT is a serine/threonine specific protein kinase that regulates various cellular processes such as glucose metabolism, apoptosis, cell proliferation transcription, and cell migration 48. On mitochondria, the PH domain and Leucine rich repeat Protein Phosphatases (PHLPP)-Akt-HK2 protein complex is easily detected. PHLPP reduces the phosphorylation of HK2, induces its separation from mitochondria, and also shuts down AKT 49. At the same time, mitochondrial translocation of HK2 protein occurs through AKT-mediated phosphorylation of HK2 47. Therefore, the absence of PTEN and the over-activation of AKT are the major carcinogenic factors in PTEN-deficient cancers. P53 can activate or inhibit many genes and regulate key processes such as the cell cycle, DNA repair and apoptosis 50. It induces the expression of the apoptosis regulator (TIGAR), reduces the level of fructose-2,6-diphosphate in cells, thereby inhibiting glycolysis and reducing intracellular ROS levels 51. And p53 can also bind directly to Glucose-6-phosphate dehydrogenase (G6PDH) and prevent the formation of active dimers, thereby inhibiting the pentose phosphate pathway 52. At the same time, it was found that p53 deletion would reduce the biosynthesis of miR143 and prevent it from degrading HK2 mRNA by binding to the three prime untranslated region, thus enhancing the stability of HK2 mRNA 44.

### 3.2. HK2-mediated glycolysis enhancement and pentose phosphate pathway

Pentose phosphate pathway (PPP) is an important glucose metabolism pathway in cells, which participates in energy metabolism and biosynthesis together with glycolysis and TCA. This process can oxidize G6P to fructose 6-phosphate or it can be non-oxidized to glyceraldehyde 3-phosphate, both of which occur in the cytoplasm 53. The main functions of PPP include cellular anti-oxidation, reduction of oxidizing products and protection of intracellular structures from oxidative damage, and the production of ribonucleic acid precursors necessary for nucleic acid synthesis.

PPP in cancer cells can synthesize pentose phosphate, which is necessary for the synthesis of nucleic acids and provides nicotinamide adenine dinucleotide phosphate (NADPH) for the synthesis of fatty acids. As a critical tumor suppressor gene, p53 is involved in negative regulation of PPP. p53 directly binds to the promoters of glucose transporter genes GLUT1 and GLUT4, inhibiting their expression, thereby suppressing glucose uptake in cancer cells and ultimately restricting both glycolysis and the PPP. p53 can also indirectly inhibit the oxidative PPP by suppressing the expression of phosphoglycerate mutase-1 (PGAM1), a glycolytic enzyme that converts 3-phosphoglycerate to 2-phosphoglycerate 54. Transketolase (TKT) is a key enzyme linking glycolysis and PPP, whose inhibition improves the efficiency of cancer treatment. For example, blocking TKT was proved to prevent the development of HCC, which demonstrated the important role of PPP in cancer development 55.

HK can phosphorylate glucose to G6P, and the resulting G6P can produce anabolic intermediates through the PPP 56, 57. G6P is a key metabolite in the PPP and an important node of intracellular glucose metabolism. As the first substrate of PPP, G6P is first catalyzed by glucose-6-phosphate dehydrogenase (G6PD), converted to 6-phosphogluconic acid (6PG), and produces NADPH. 6-phosphogluconate dehydrogenase then catalyzes the conversion of 6PG to D-lactate-6-phosphate (D-6PG), which yields NADPH. Subsequently, D-6PG is catalyzed by a series of enzymes, such as D-lactate-6-phosphate dehydrogenase, 3-phosphoglycerate kinase, phosphoglycerate mutase, phosphoglycerate kinase, and phosphopentose isomerase, to gradually transform into other intermediate products, and finally to form G6P again, forming a cycle 54, 58, 59.

It was found that HK2 and G6PDH were overexpressed in cancer-associated adipose tissue, accompanied by increasing PPP activity 60. Meanwhile, in malignant breast cancer cells, 5' AMP-activated protein kinase (AMPK) and HK2 protein levels increased, while downstream proteins phosphofructokinase-1 (PFK-1) and GAPDH levels did not change, suggesting that metabolic reprogramming and increased glucose utilization in tumor-associated adipose tissue were mainly targeted at the PPP 60. It has also been suggested that HK2 provides G6P for glycogen synthesis and lipid synthesis through PPP 1. This suggests that the action of HK2 is limited by many factors. When glucose is lacking, HK2 will locate in the cytoplasm and promote G6P to enter the glycogen synthesis and PPP. When HK2 binds to mitochondria, G6P is preferentially subjected to glycolysis and oxidative phosphorylation 8, 16. At the same time, it has also been found that the increased activity of PPP might further enhance the expression of HK2 by stabilizing BACH1 5.

### 3.3. HK2-mediated glycolysis enhancement and glutamine metabolism

By enhancing glycolysis, cells are able to improve ATP synthesis, which provides energy for glutamine synthesis, while promoting NADPH production and maintaining the formation of glutamine reduction. Glutamine, a vital intracellular energy substrate, is believed to hold significant importance in cancer cells due to its higher consumption compared to synthesis within these cells 61. Glutamine not only provides a nitrogen source for amino acid and nucleotide biosynthesis in cancer cells, but also is a carbon source for supplementing the TCA cycle and lipid biosynthesis pathways 62. Glutamine-derived α-ketoglutarate can be used as a supplement substrate to re-drive the inhibited TCA cycle in tumor cells 63. The study found that glutamine deficiency caused Triple-negative breast cancer (TNBC) cells to concentrate in the S phase of the cell cycle, making them unable to survive or proliferate 64.

It has been demonstrated experimentally that in non-small cell lung cancer cells, the decrease of HK2 expression was accompanied by the downregulation of glutaminase (GLS) expression, and the cellular glutamine metabolism was weakened 65. GLS plays a crucial role in cellular glutamine metabolism, catalyzing the hydrolysis of glutamine into glutamate and ammonia, or the synthesis of glutamine by combining glutamate with ammonia 66. These reactions not only participate in the regulation of cellular nitrogen metabolism, but also are involved in the process of intracellular energy production and cell proliferation. The activity of GLS affects intracellular glutamine levels and their associated metabolic pathways and is therefore essential for maintaining intracellular balance and normal function. Furthermore, when glutamate acts outside pancreatic ductal adenocarcinoma (PDAC) cells, it can induce calcium influx in PDAC cells through N-methyl-D-aspartate receptor (NMDAR), which then activates downstream Ca^2+^ dependent protein kinase calmodulin-dependent protein kinase II (CaMKII)/extracellular signal-regulated kinases (ERK)-mitogen-activated protein kinase (MAPK) pathway and promotes transcription of METTL3 gene. Subsequently, METTL3 upregulates the expression of HK2 by modifying m^6^A in mRNA 67.

### 3.4. HK2-mediated glycolysis enhancement and mitochondrial function

There is a close interaction between HK2-mediated glycolysis enhancement and mitochondrial function. Common mitochondrial gene mutations in cancer cells alter mitochondrial bioenergy and biosynthesis, initiate retrograde signalling with the nucleus, and regulate signalling pathways and chromatin structure to suit the needs of cancer cells. Cancer cells then reprogram neighbouring stromal cells to optimise their environment, enabling rapid growth and survival 68. Metformin exerts its inhibitory effect on the hydrophobic interface of mitochondrial complex I by reversibly binding to functionally crucial hydrophilic regions. This biguanide compound, commonly prescribed for diabetes management, has demonstrated antitumor properties in diabetic patients 69. The inhibition of mitochondrial complex I by metformin, leading to mitochondrial dysfunction, may be a key factor in its potential anticancer properties. This suggests that mitochondrial function is closely related to cancer cell growth.

In the case of normal blood sugar, 70-80% of HK2 combined with mitochondria, promote aerobic glycolysis 8. Therefore, this may only be seen in cancer cells, rather than a general rule for “body cells.” The binding site of HK2 to mitochondria is Mitochondria-associated ER membrane(MAM), The site where HK2 binds to mitochondria is the MAM, where VDAC is also located 70. The VDAC family, also known as the voltage-dependent ion channel family, includes VDAC1, VDAC2, and VDAC3. They are the primary protein channels found in the outer membrane of the mitochondria and play a role in controlling cell metabolism and mitochondrial function, particularly in the pathways leading to apoptosis and energy consumption 71. The MAM is an important intracellular subcellular structure that serves as the interface between endoplasmic reticulum (ER) and mitochondria, is enriched in a variety of proteins including ER membrane proteins, mitochondrial outer membrane proteins and specific lipid components, and is involved in the regulation of important cellular processes such as apoptosis, autophagy, inflammation and inflammasome formation 72, 73. Importantly, research has revealed that the dissociation of HK2 from MAM could trigger Ca^2+^ influx into mitochondria, resulting in mitochondrial depolarization and calcium protease-mediated cell death 70. Mitochondria are under the regulation of Ca^2+^ signaling in various activities 74, and utilizing calcium as an important second messenger, regulating the proliferation of cancer cells, metastasis and invasion. HK2 is involved in calcium stabilization, and it is conceivable that HK2 can maintain the health of cancer cells and fight cell death.

Numerous investigations have also revealed that the binding of HK2 to VDAC1 could inhibit the binding of Bcl-2-associated X-protein (BAX) to VDAC1, hinder the translocation of BAX from the cytoplasm to the mitochondria, and obstruct BAX's release of cytochrome C, all of which could prevent apoptosis and mitochondrial malfunction 8, 20, 75, 76. HK2 phosphorylation by AKT increase its binding to mitochondria, favoring cell survival 77.

**Figure 2:**
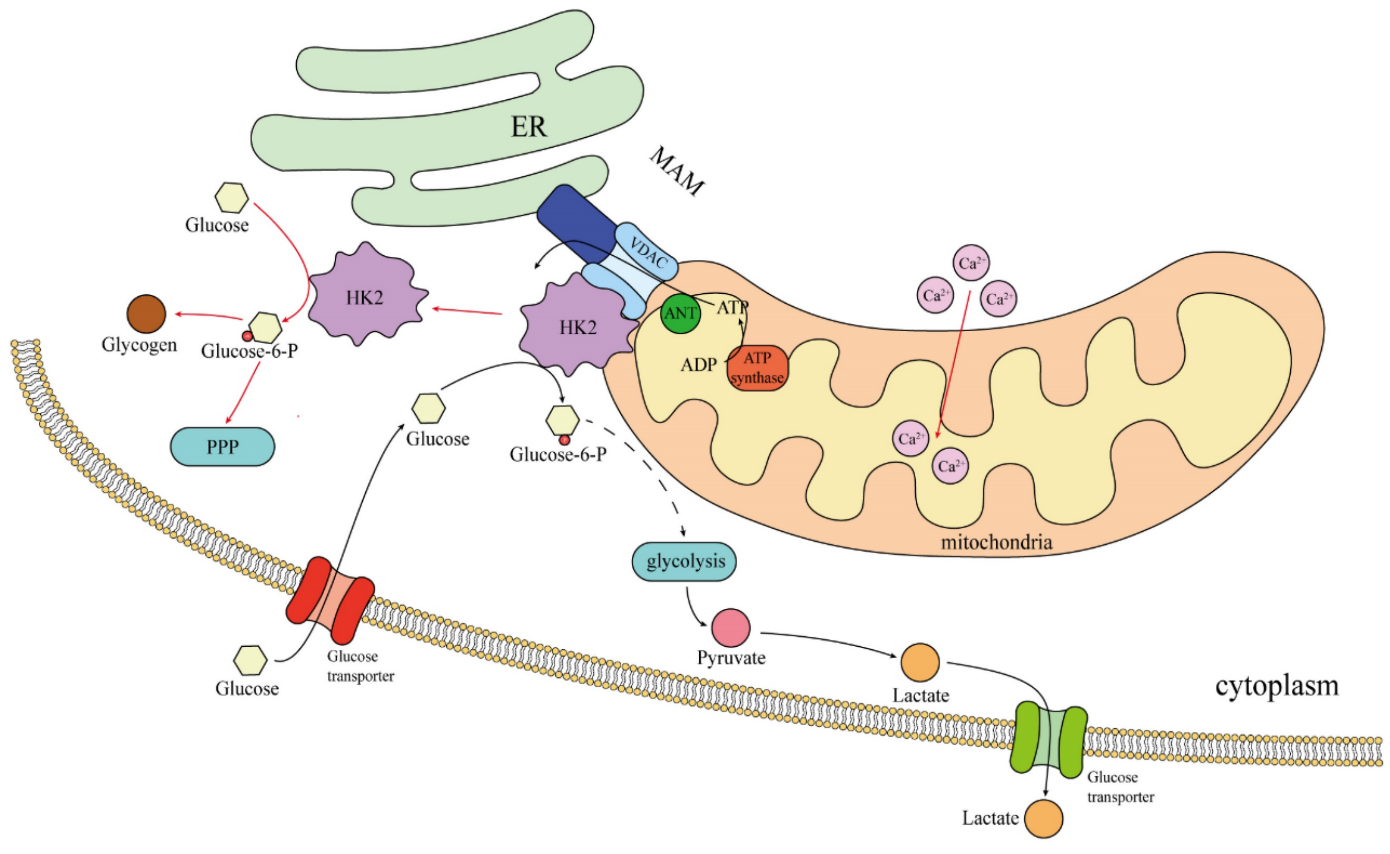
Localization and regulation of HK2 in tumor cells. The black arrow indicates the presence of glucose in the cell; The red arrow indicates the removal of extracellular glucose.

To summarize, upon binding to mitochondria, HK2 engages in interactions with enzymes from there, including lactate dehydrogenase and glucose phosphatase. This enhances the relationship between the glycolytic pathway and mitochondria, diminishes the effectiveness of oxidative phosphorylation of mitochondria **(Fig. 2)**.

### 3.5. HK2-mediated enhancement of glycolysis and reactive oxygen species

Reactive oxygen species (ROS) are a class of highly active oxidizing molecules, including superoxide anions, hydrogen peroxide, and hydroxyl radicals, which are by-products of the electron transport chain of mitochondria. In the human body, ROS is a normal cell metabolite, but can also be overproduced in response to exogenous stimuli. Appropriate ROS plays an important role in the physiological processes of cell signaling, immune response and apoptosis. However, too much ROS can damage proteins, lipids and nucleic acids, leading to oxidative stress, and the elevation of ROS in mitochondria can induce cell apoptosis 78. Current studies have found that chemotherapy drugs such as platinum derivatives and gemcitabine could cause oxidative DNA damage in tumor cells by producing high levels of ROS 79.

Hexokinase in rat brain mitochondria reduces mitochondrial ROS production through the ADP cycle mechanism 80. The study found that knocking down HK2 in cardiomyocyte might affect mitochondrial permeability and thus increased ROS production, and this situation has been confirmed *in vivo* and *in vitro* 81. Under hypoxia, HK2 forms complexes with TIGAR on the mitochondrial surface, thereby reducing mitochondrial ROS levels and promoting cell survival 78. More experiments have found that HK2 on mitochondria limited the production of mitochondrial ROS by maintaining local ADP levels 80.

### 3.6. HK2-mediated glycolysis enhancement and lactic acid production

There is a close relationship between HK2 -mediated enhancement of glycolysis and lactic acid production. By phosphorylating glucose to G6P, HK2 promotes the initiation of the glycolytic pathway, nourishing the energy and biosynthetic substances needed for rapidly proliferating cancer cells 82-84. Moreover, as an end product of glycolysis, lactic acid accumulates in large quantities in the tumor microenvironment, affecting the acidity level of the tumor microenvironment, slowing down the function of local immune cells, and promoting the growth and spread of tumor cells 82. At the same time, lactic acid can inhibit the activity of T cells, interfere with the anti-tumor immune response, and promote the development of tumors 85. Lactic acid is also a natural inhibitor of the tumor suppressor p53 86. Serum lactate concentration was measured in 140 patients with different malignant tumors, and it was found that about one-third of patients with high tumor load had significantly higher serum lactate levels than patients with low burden or remission, which confirmed the fact that tumors produce large amounts of lactate 87. At the same time, lactic acid and its mediated activation of GPR81 signaling pathway are particularly important in the development of cancer, such as promoting cell proliferation, invasion, promoting angiogenesis, immune escape and generating chemical resistance 88. Studies have found that lactic acid, as an autocrine signaling molecule in tumor cells, can promote the expression of GPR81 receptor and produce an oncogenic phenotype 89. Lactic acid also induces a previously unknown post-translational modification (PTM) called lactation. High MRE11 lactation can promote HR and chemical resistance of cancer cells, whereas inhibition of MRE11 lactation can weaken HR and promote the sensitivity of cancer cells to chemotherapy 90. Dichloroacetic acid (DCA) can inhibit pyruvate dehydrogenase kinase and reduce lactic acid production 91. Existing Phase I clinical trials have demonstrated that oral DCA was feasible and well tolerated in patients with recurrent glioblastoma and brain metastases 92.

Since HK2 is strongly associated with increased lactic acid production, inhibiting HK2 expression may help reduce lactic acid production. Experiments aimed at silencing HK2 in human embryonic kidney cells, liver cancer cells, mouse mononuclear macrophages, and immortalized bone marrow macrophages have demonstrated a reduction in glycolysis as indicated by decreased levels of pyruvate and lactic acid 93. Additionally, it was discovered that mitochondrial antiviral-signaling (MAVS) knockdown and deletion cells displayed lower amounts of pyruvate and lactic acid, as well as a lowered extracellular acidification rate (ECAR), in comparison to normal cells. MAVS was reported to bind with HK, thus influencing its mitochondrial localization and activity 93. There are also studies using albuminol to inhibit the expression of HK2 in liver cancer cells, resulting in reduced glucose uptake and lactic acid production in liver cancer cells 94, 95. In the nude mouse model of subcutaneous tumor, the increase of HK2 mRNA was accompanied by the increase of lactic acid level 96.

## 4. Advances in natural product research with HK2 as therapeutic target

**Figure 3:**
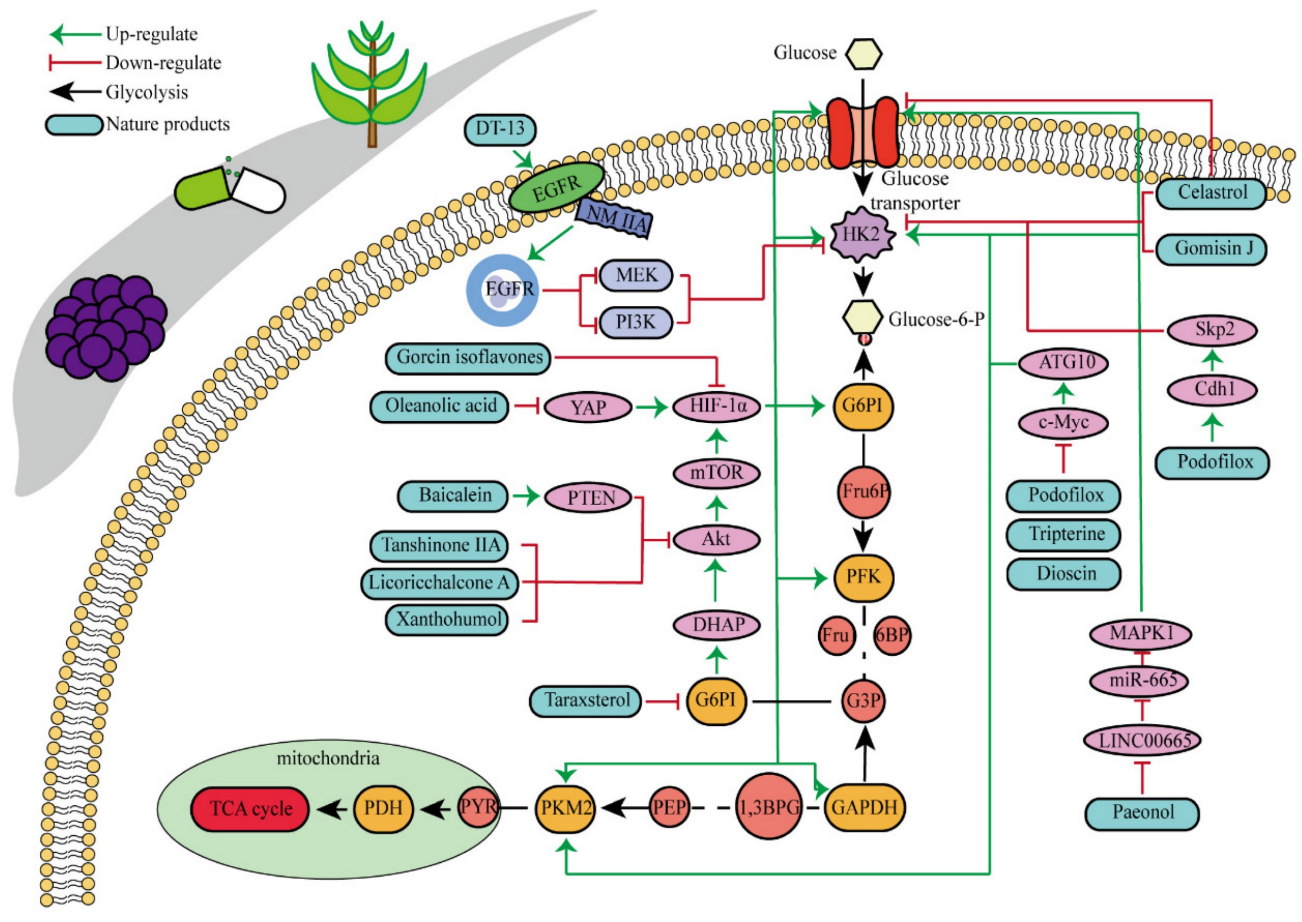
Natural products that inhibit HK2 activity and their detailed mechanisms.

Natural products are organic compounds derived from plants, animals and microorganisms, with diverse chemical structures and biological activities, which play a key role in the treatment of various diseases including cancer 97. And many studies have indicated that HK2 was a direct target of some natural products with anticancer properties, or that the expression and activity of HK2 could be indirectly influenced by certain natural products. Multiple natural products have been identified to directly or indirectly inhibit HK2 for their anticancer effects, as illustrated in **Fig. 3** and **Table 1**. Key components encompass flavonoids, phenols, lignans, and pentacyclic triterpenes, among others.

Baicalein is a kind of natural flavonoid which mainly exists in *Scutellaria baicalensis,* and shows effects on inhibiting cell proliferation and inducing cell apoptosis 98. Related experiments have found that baicalein, the aglycone of baicalein inhibited hypoxia-enhanced glycolytic flux in AGS cells, and reduced the expression of key glycolytic-related enzymes such as HK2, lactate dehydrogenase A (LDH-A) and pyruvate dehydrogenase lipoamide kinase isozyme 1 (PDK1) 99. Baicalein can play an anticancer role by regulating various cell signaling pathways, such as the PI3K/AKT pathway and the PTEN/AKT/HIF-1α signaling pathway 98-100. Baicalein can also inhibit hypoxia-induced AKT phosphorylation by enhancing PTEN accumulation 98. Licochalcone A is a flavonoid extracted from *Licorice*, possessing anticancer, antioxidant, anti-inflammatory, and antiviral properties 101. Both *in vivo* and *in vitro* experiments showed that licochalcone A could reduce HK2 expression, inhibit glucose consumption and lactic acid production in gastric cancer cells, and hinder cell survival and proliferation 102. Licochalcone A can inhibit a variety of signaling pathways such as AKT, ERK and NF-κB. It was previously thought that the inhibition of licochalcone A on glycolysis might be mainly attributed to the blocking of AKT signaling pathway 102.

Morusin is a natural product that can be extracted from plants of the mulberry genus, such as mulberry. As a flavonoid, morusin has shown antibacterial, anti-inflammatory and anti-tumor activities 103. Studies have found that morusin could effectively reduce the expression of HK2 in liver cancer cells, inhibit cell glycolysis, thereby impeding cell proliferation and reducing cellular activity 104. Chrysin is a bioactive flavonoid found mainly in passionflower, honey and propolis. Chrysin have showed a variety of pharmacological activities, including antioxidant, anti-inflammatory and anticancer properties 105. Hepatocellular carcinoma cells with high expression of HK2 were treated with chrysin, and cell proliferation and glycolysis were inhibited, while HK2 content in mitochondria was significantly reduced. However, when HK2 was overexpressed in cells, the inhibitory effect of chrysin on glycolysis and apoptosis was hindered. Researches have recovered that the decrease of HK2 leaded to a sharp increase of BAX in mitochondria, which promoted the binding of BAX and VDAC to form a VDAC-BAX complex to induce apoptosis, which might also be the mechanism of inhibiting the proliferation of cancer cells by chrysin 106.

Hops are specific to a substance called xanthohumol, which belongs to a group of isopentene flavonoids. It has antioxidant, anti-inflammatory and potential antitumor activities 107. In human colorectal cancer cells, xanthohumol significantly down-regulates glucose consumption and lactic acid production of cells, and inhibits cell proliferation. Mechanism study suggested that treatment with xanthohumol decreased HK2 protein content and inhibited activation of AKT in colorectal cancer cells. Therefore, the authors believed that xanthohumol might inhibit HK2 and glycolysis in colorectal cancer cells through the inhibition of AKT. *In vivo* experiments have also verified that xanthohumol could inhibit the proliferation of tumor cells 108. Genistein is a kind of natural plant compound, belonging to the isoflavone family. It is mainly found in some plants, such as broom and red clover. Studies have shown that genistein had many biological activities such as antioxidant, anti-inflammatory, and anticancer 109. As reported, the proliferation of hepatocellular carcinoma cells was inhibited, cell cycle was stagnated, and glycolysis was inhibited after genistein treatment. Genistein inhibited HIF-1α protein expression and transcriptional activity, by which the contents of GLUT1 and HK2 in liver cancer cells were down-regulated. In mice, genistein can also effectively inhibit the growth of liver cancer tissue 110.

Oleanolic acid is one of the main components of tea leaves and tea fruits, and is also widely present in other plants. As a pentacyclic triterpenoids, oleanolic acid has antioxidant and anti-inflammatory properties 111. Studies have showed that Oleanolic acid could decrease the nuclear abundance of HIF-1α, down-regulate the expression of HIF-1α in human gastric cancer cells, and down-regulate the expression and activity of the glycolytic rate-limiting enzymes PFK-1 and HK2 25, 112. Celastrol and triptolide are extracted from plants of the triptolide family, of which celastrol is a pentacyclic triterpenoid compound and triptolide is a pentacyclic diterpenoid compound. Both are widely used in traditional Chinese medicine and modern medicine and have multiple biological activities such as anti-inflammatory, immunomodulatory, anti-tumour and antioxidant 113. It has been found that celastrol could inhibit the expression and activity of HK2 in gastric cancer cells, thus inhibiting their glycolysis 25. In head and neck cancer cells, triptolide treatment can reduce HK2 protein levels and inhibit the binding of HK2 to mitochondria. At the same time, by inhibiting mitochondria-associated HK2, triptolide can also activate the Bcl-XL/Bcl-2-associated death promoter (BAD)/BAX-caspase 3-GSDME cascade, thus promoting cell death 114. Dandelion sterol is a pentacyclic triterpenoid extracted from dandelion and is widely utilized in medicine and healthcare. It possesses various biological activities including hypoglycemic, antioxidant, anti-inflammatory, and immunomodulatory properties 115, 116. Dandelion sterol could decrease HK2 expression and inhibit glycerol 3-phosphate dehydrogenase 2 (GPD2) -mediated glycolysis in gastric cancer cell line HGC-27, but the specific reduction pathway was not clear 117.

Paeonol can be found in various common Chinese herbal medicine, which exhibits antibacterial, anti-inflammatory, antioxidant and anti-tumor activities 118. Paeonol could reduce HK2 levels and inhibit glycolysis by regulating LINC00665/miR-665/MAPK1 axis, which was confirmed in gastric cancer cells 25, 119. Salidroside, a phenolic compound extracted from the Chinese herb *Rhodiola rosea*, is widely used in pharmacology and clinical medicine and is known for its remarkable antioxidant, anti-inflammatory, neuroprotective and anti-tumor properties 120. The study showed that in stomach cancer mouse model cells, 50 mg/kg/d of salidroside could lower the expression levels of the HK2 and GLUT1 proteins 121.

Podofilox, a lignan derived from the rhizome of *Podophyllum*, and its derivatives exhibit promising therapeutic effects on cancer. Studies have shown that podofilox could interfere with the microtubule structure within tumor cells, thereby preventing cell division, inducing apoptosis, and inhibiting tumor growth 122. Experiments demonstrated that podofilox inhibited colony formation and decreased levels of HK2, PKM2, c-Myc and autophagy related 10 (ATG10) in gastric cancer cell lines AGS and HGC-27 123. Gomisin J is a small molecular weight lignan compound found in *Schisandra chinensis*. Studies have shown the antioxidant, anti-inflammatory and anti-tumor biological activities of Gomisin J. This compound has also shown potential benefits for neuroprotection, cardiovascular health and liver protection 124. It was found that glioma cells treated with Gomisin J exhibited mitochondrial dysfunction, decreased glucose uptake, inhibited cell proliferation, and decreased HK2 expression. Gomisin J effectively inhibited the binding of HK2 and VDAC in mitochondria. Overexpression of HK2 could significantly restore mitochondrial function and glucose uptake ability of Gomisin J treated glioma cells 125.

DT-13 is a modified bioactive peptide derived from venomous snakes and has significant antitumor effects. Studies have shown that DT-13 could inhibit the growth of various cancer cells by inducing apoptosis, inhibiting tumor proliferation and angiogenesis 126. The combination therapy of DT-13 and topotecan has shown great potential in cancer treatment, exerting multiple effects on HK2 including its activity, expression level, and binding to mitochondria. The induction of HK2 inactivation primarily occurs via non-muscle myosin IIA. The reduction in HK2 expression is mainly due to the inhibition of the mitogen-activated protein kinase kinase (MEK) and PI3K pathways of the epidermal growth factor receptor (EGFR), which subsequently affects the AMPK-mTOR pathway. The specific binding of HK2 to mitochondria is inhibited, manifested by decreased HK2 expression in mitochondria, reduced co-localization of HK2 with mitochondria, leading to a weakening of aerobic glycolysis 127. Dioscin is a kind of saponin compound which mainly exists in Chinese yam. It has a variety of pharmacological activities, including anti-inflammatory, antioxidant, anti-tumor and immunomodulatory effects 128. *In vitro* studies have shown that dioscin can inhibit the proliferation of colorectal cancer cells and inhibit their glycolysis. After treatment, the expression of HK2 in the cells was decreased. The interaction between HK2 and VDAC1 was also found to be significantly. Mechanistically, dioscin decreased the expression of transcription factor c-Myc, which is involved in the regulation of HK2. *In vivo* studies showed that dioscin inhibited the growth of colorectal cancer xenografts in nude mice 129. Additionally, some scholars have found that knocking out S-phase kinase-associated protein 2 (Skp2) could inhibit HK2 expression and glycolysis, and thereby limit the growth of colorectal cancer cells *in vivo* and *in vitro*. Dioscin can decrease HK2 protein levels in colorectal cancer cells and promote Skp2 degradation in a ubiquitination-dependent manner 130. Tanshinone IIA is a bioactive ingredient extracted from salvia miltiorrhiza with many biological activities such as anticoagulation, antioxidant, anti-inflammatory and cardiovascular protection 131. In recent years, there has been a certain level of attention on the potential of Tanshinone IIA in tumor treatment. In oral squamous cell cancer cells (OSCC), tanshinone IIA treatment reduced glucose consumption and lactic acid production of the cells, and decreased cell viability and increased the number of apoptosis cells. Mechanism research has revealed that among numerous glycolytic enzymes, HK2 was the most impacted by tanshinone IIA, with its transcription reduction ranking highest. Overexpression of HK2 was used to investigate its role in tanshinone IIA-induced apoptosis. Further studies found that tanshinone IIA promoted F-box and WD repeat domain containing 7 (FBW7)-mediated c-Myc destruction, thereby inhibiting HK2 transcriptional expression. The results of *in vivo* experiments in mice also indicated that tanshinone IIA inhibited tumor development of OSCC cells in mice 132. The pharmacological processes of these natural compounds remain poorly known despite a large number of studies having been conducted; hence, more study is required.

## 5. Summary and prospects

HK2 is involved in the regulation of glycolytic pathways, and providing energy and biosynthetic substances for tumor growth and invasion. HK2 catalyzes the conversion of glucose into G6P, a key metabolite in the glycolysis, PPP and glycogen synthesis, and influences the growth and metabolism of tumor cells by regulating the transformation of fructose phosphate. HK2 expression is associated with GLS expression and cellular glutamine metabolism. Mitochondria are complex organelles with multiple functions, and HK2 can bind to outer membrane of them though VDAC. It can change mitochondrial membrane permeability, damage mitochondrial function, affect ROS levels, inhibit mitochondrial apoptosis pathway, and ultimately promote the survival and proliferation of tumor cells. HK2 also promotes the accumulation of lactic acid, affects the acidity level of the tumor microenvironment, and slows down the immune effect. These multiple activities allow HK2 to play a key role in the growth and survival of tumor cells.

Targeting HK2 has emerged as a promising therapeutic approach. Numerous natural products that have demonstrated significant anticancer activity have also been found to inhibit HK2 levels or activity. However, many studies use HK2 merely as a simple detection marker. It remains unclear whether these natural products directly inhibit HK2 expression, promote its degradation, or affect only its enzymatic activity without altering its levels. Elucidating these detailed molecular mechanisms is crucial for the further development of HK2 inhibitors with anticancer activity, thus marking a key direction for future research.

Taken together, HK2 can provide the energy needed by cancer cells, mediate cell survival, inhibit apoptosis. Its role in tumorigenesis and development, and the promise of targeted therapies are encouraging. With the overcoming of current challenges and more in-depth cell and animal model studies and even clinical studies, the targeted therapy of HK2 is expected to become an important strategy for tumor therapy, bringing more treatment options to patients. Identifying HK2 inhibitors from natural products and elucidating their mechanisms of action can offer a wider selection of lead compounds for anticancer drug development. These natural products may inhibit HK2 through unknown molecular mechanisms, potentially providing novel strategies for drug development.

## Figures and Tables

**Table 1:** Specific information on the anticancer effects of natural products that inhibit HK2.

Compounds	Chemical structures	Cancer types	*In vitro* experiments	*In vivo* experiments	References
Baicalein	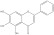	Gastric adenocarcinoma	AGS cells (10, 20 and 40 µM)		99
Licoricchalcone A	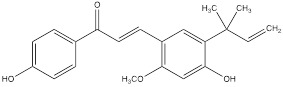	Gastric cancer	MKN45 cells (60 µM) SGC7901 cells (60 µM)	BALB/ca nude mouse model of gastric cancer (10 mg/kg)	102
Morusin	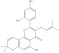	Liver cancer	Hep3B cells (0-40 μM) Huh7 cells (0-40 μM)		104
Chrysin	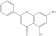	Liver cancer	HepG2 cells (0-60 µM) Hep3B cells (0-60 µM) Huh-7 cells (0-60 µM) HCC-LM3 cells (0-60 µM) Bel-7402 cells (0-60 µM) SMMC-7721 cells (0-60 µM)	Six-week-old female nu/nu thymus free nude mouse model of liver cancer (30 mg/kg)	106
Xanthohumol	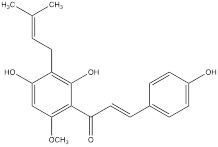	Colorectal cancer	HT29 cells (0, 2, 4, 8 μM) SW480 cells (0, 2, 4, 8 μM) LoVo cells (0, 2, 4, 8 μM) HCT116 cells (0, 2, 4, 8 μM) SW620 cells (0, 2, 4, 8 μM)	Seven-week-old female nude mouse model of colorectal cancer without thymus (10 mg/kg)	108
Genistein	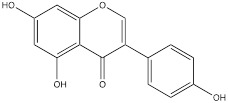	Liver cancer	HCC-LM3 cells (0-80 μM) SMMC-7721 cells (0-80 μM) Hep3B cells (0-80 μM) Bel-7402 cells (0-80 μM) Huh-7 cells (0-80 μM)	Four-week old male thymus free BALB/C nu/nu mouse model of liver cancer (20, 40 and 80 mg/kg)	110
Oleanolic acid	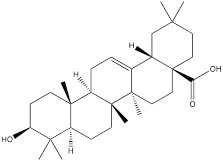	Gastric cancer	MKN-45 cells (30 μM) SGC-7901 cells (30 μM)		112
Celastrol	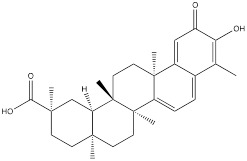	Gastric cancer	BGC-823 cells (0.75, 1.0, 1.5 μM)		25
Triptolide	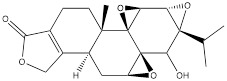	Head and neck cancer	HK1 cells (50 nM) C666-1cells (50 nM) FaDu cells (50 nM)	Five-week old male thymus free BALB/C nu/nu mouse model of head and neck cancer model (0.1 mg/kg)	114
Dandelion sterol	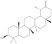	Gastric cancer	HGC-27 cells (15 µM)		117
Paeonol	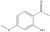	Gastric cancer	BGC-823 cells (60 µg/mL) MGC-803 cells (60 µg/mL)	Female BALB/c nude mouse model of gastric cancer (30 mg/kg, 50 mg/kg)	119
Salidroside	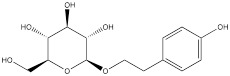	Gastric cancer	SGC-7901 cells (80 µM) MKN-45 cells (80 µM)	BALB/c nude male mouse model of gastric cancer (50 mg/kg)	121
Podofilox	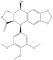	Gastric cancer	AGS cells (3.4 nM) HGC-27 cells (3.4 nM)		123
Gomisin J	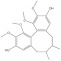	Glioma	U87 cells (0-100 μM) U251 cells (0-100 μM)	Four-week-old male BALB/C nude mouse model of glioma (40 mg/kg)	125
DT-13	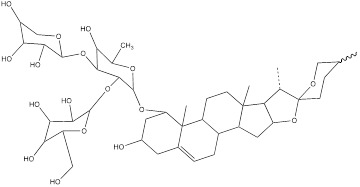	Gastric cancer	BGC-823 cells (10 μM)	BALB/c nude mouse model of gastric cancer without thymus (0.625 mg/kg)	127
Dioscin	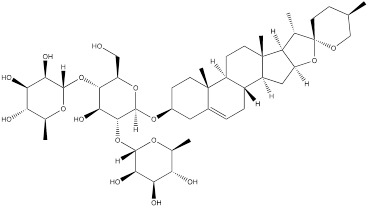	Colorectal cancer	HCT116 cells (0-5μM) HT29 cells (0-5μM) DLD1 cells (0-5μM) SW620 cells (0-5μM) SW480 cells (0-4μM) HCT8 cells (0-4μM)	Female nude mice model of colorectal cancer (5 mg/kg) Six-week-old nude mouse model of colorectal cancer without thymus (10 mg/kg)	129, 130
Tanshinone IIA	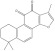	Oral squamous cell carcinoma	SCC9 cells (0-5 μM) SCC15 cells (0-5 μM) SCC25 cells (0-5 μM) CAL27 cells (0-5 μM)	Six-week-old nude mice model of oral squamous cell carcinoma without thymus (10mg/kg)	132
